# The power of ESG in shaping dividend policy: Illuminating the role of financial sustainability in an emerging market

**DOI:** 10.1371/journal.pone.0312290

**Published:** 2024-12-05

**Authors:** Abdulateif A. Almulhim, Abdullah A. Aljughaiman, Thamir Al Barrak, Kaouther Chebbi, Nagwa Amin

**Affiliations:** 1 Finance Department, Associate Professor of Finance, School of Business, King Faisal University, Al Ahsa, Saudi Arabia; 2 Accounting Department, Assistant Professor of Accounting, School of Business, King Faisal University, Al Ahsa, Saudi Arabia; 3 Economic Department, Assistant Professor of Economic, School of Business, King Faisal University, Al Ahsa, Saudi Arabia; Universitat Jaume I, SPAIN

## Abstract

This study investigates the impact of environmental, social, and governance (ESG) scores on dividend policy, while taking into account the moderating effect of financial sustainability. It examines data from companies listed on the Saudi Exchange, during the period spanning the years from 2013 to 2022. According to the findings of panel regression analysis, there is a strong positive correlation between ESG performance and dividend payments. In essence, businesses that exhibit strong ESG practices continuously maintain dividend payments as a way of demonstrating their dedication to both stakeholders and shareholders. Furthermore, financial sustainability exerts an enhancing influence on the ESG–dividend relationship, indicating that the positive effect of ESG on dividend yields is significant in financially sustainable companies compared to their peers. It is noteworthy that these conclusions hold up well even when put through sensitivity studies using different estimating methods. The implications of these results extend to a broad spectrum of stakeholders, including investors, management, analysts, and policymakers. They provide valuable insights for companies and markets seeking to expand their ESG initiatives.

## 1. Introduction

Historically, the main goal of investment in companies has been to produce a return on that investment by means of dividends. The factors that influence dividend payouts and how they connect to financial and governance factors have been the subject of numerous research studies and theories [1,2]. However, there has been a substantial change in business operations as a result of the recent global trend toward addressing environmental and social issues. This change has caused corporations to reassess how they allocate their resources in favor of policies that align environmental, social, and governance (ESG) principles that are valued by both shareholders and the general public at large [3].

This change has created new challenges for management and investors, reconfiguring company goals and establishing environmental programs as priorities, frequently to the detriment of shareholder interests. As a result, a financial dilemma emerges, as businesses attempt to strike a balance between their need to pay dividends to investors and their capacity to invest in ESG projects [4].

A number of studies have recently examined critically how companies’ approaches to dividend distribution are impacted by their alignment with ESG goals [4–7]. These investigations have yielded a range of findings, some of which have shown a positive impact of ESG ratings on a company’s capacity to distribute dividends, while others have discovered a negative association.

In this respect, beneficial associations have been noted across a range of international contexts, including the European Union [8], the United States [5], Korea [9], France [10], the United Arab Emirates [11], South Africa [12], and Western Europe [13]. However, multiple studies in China, including [6–15], have identified negative connections between ESG variables and a company’s capacity to distribute dividends, identifying a counter-narrative to the findings of these studies.

In Saudi Arabia, companies are rapidly recognizing the value of ESG disclosure. This recognition has been fueled by the implementation of governmental frameworks aimed at increasing transparency and implementing environmental practices. For instance, Saudi Arabia launched a major national sustainable development program known as the Saudi Vision 2030 in 2016. This bold plan also recognized the significance of tackling ESG problems [16,17]. The Saudi Vision 2030 recognizes the critical significance of corporate sustainability not only for the well-being of current and future generations, but also for the general welfare of the population [18]. In 2017, the Saudi Capital Market Authority (CMA) announced significant amendments to the Saudi Corporate Governance Code (SCGC) in accordance with the objectives established in the Saudi Vision 2030. These modifications included SCGC Articles 87 and 88, which, in particular, address ESG issues. These modifications have included significant ESG provisions. As a result, Saudi enterprises are now required to publish their ESG operations in accordance with the amended SCGC. Moreover, the CMA went a step further in 2018 by becoming a partner in collaborative interaction with the United Nations "Sustainable Stock Exchanges Initiative" (Capital Market Authority ESG Disclosure Guidelines (2022).

In accordance with this pledge, in October 2021, the CMA issued the "ESG Disclosure Guidelines, 2022". These Guidelines provide a helpful resource for Saudi publicly traded companies, guiding them in navigating the complex world of ESG problems. According to a recent study, the Guidelines offer issuers an overview of ESG, highlight crucial considerations for companies, and initiate a range of reporting options that enable businesses to initiate their ESG endeavors and measure their ESG initiatives. Finally, it should be noted that Saudi Arabia has made considerable progress in effectively integrating ESG principles across the Middle East and North Africa (MENA) region. According to another survey [19], the country came second in this category, demonstrating its commitment to ESG concepts and strategies. Thus, drawing on institutional, stakeholder, and legitimacy theories, the main goal of this study is to explore corporate dividend distribution strategies within the context of ESG considerations. This examination will be conducted from the angle of the conventional examination of dividends as payouts and is carried out within the distinctive context of Saudi Arabia.

Furthermore, it is essential to consider moderating factors, like corporate governance, which have been shown to wield a significant influence on the connection between ESG factors and dividend policies, as demonstrated by research studies [11–20]. The role of intermediary factors within ESG strategies, such as efficiency, costs and activities, is also a significant consideration, as discussed in the research [21]. Elements such as firm short-term and long-term value come into play, impacting ESG performance and dividend distribution policies. These dynamics can vary across different cultural and regional contexts. As a result, the second purpose of our study is to investigate the moderating effect of corporate financial sustainability in the ESG–dividend association, with an emphasis on Saudi Arabia, a region that serves as a model for ESG practices, compliance, and reporting standards in emerging markets.

For this purpose, our research relies on information collected from 332 company-year data points spanning the years from 2013 to 2022. We have detected that ESG is significantly and favorably correlated with the dividend policy. This result is attributed to the fact that transparent disclosure furnishes shareholders with precise insights into the company’s cash flow generation, enabling them to make more informed decisions regarding dividends. In addition, paying dividends remains a key focus, and engaging in ESG practices does not negate the importance of rewarding shareholders. Instead, it serves to mitigate agency conflicts, diminish information imbalances, and sends favorable signals to financial markets [4–10]. In addition, we found that corporate financial sustainability enhances the positive impact of ESG on dividend payouts. Financially sustainable businesses are becoming more prevalent. The ESG practices are being purposefully and strategically invested in by these businesses. According to [22], this dedication to ESG is more than just a fad, it is a deliberate attempt to build a favorable reputation and foster confidence among stakeholders. At the same time, for businesses with strong financial resilience, the payment of dividends to investors remains a top priority.

The practical insights gained from this study could assist the Saudi financial authorities in incorporating ESG disclosure into their reporting procedures; thereby, increasing openness among listed companies in Saudi Arabia’s financial markets. It is worth noting that, as far as we know, no previous research has looked into the influence of ESG disclosure on dividend payouts. This study fills that need by providing useful evidence about the impact of ESG activities on dividend yields, emphasizing the critical role of financial sustainability in creating this relationship.

This study contributes to the existing literature in several ways. We add to the ESG literature [23–27] and dividend studies [28,29] through investigating the influences of ESG on a firm’s payout level. Previous studies focusing on ESG have unveiled a perplexing landscape characterized by inconsistencies and contradictions in the association between ESG and financial performance. These discrepancies are not limited to theoretical aspects, such as legitimacy, stakeholder engagement, and agency theory, but also extend to empirical observations [22]. Prior research has failed to establish agreement regarding the impact of ESG and financial performance [30]. Consequently, the incongruities, both in theoretical foundations and empirical findings, surrounding this relationship remain a subject of debate, emphasizing the pressing necessity for additional investigations in this realm.

Unlike previous studies [31,32], which only examine the influence of one aspect such as environmental sustainability or the social environment on dividend policy, we consider a comprehensive picture of sustainability indictors, environment, social, and governance, and their impact on corporate payouts. Thus, this study highlights the important role that ESG disclosure plays in business. ESG disclosure is a concrete example of a company’s commitment to social responsibility, environmental stewardship, and increased transparency. This study examines it not in isolation but rather as a fundamental part of a range of corporate policies.

Furthermore, we also contribute to the previous literature by examining the moderating effect of financial sustainability on the association between ESG and corporate payouts. To the best of our knowledge, no study has examined the moderating effect of corporate financial sustainability on the association between ESG activities and financial dividend yields. To date, there has been a notable absence of substantial evidence underscoring the imperative for companies to embrace and integrate ESG principles into their strategic planning and operational frameworks [33]. This paper is also the first to introduce a fresh viewpoint on ESG activities by scrutinizing companies situated in an emerging market within the GCC (Gulf Cooperation Council) area. This region is typically perceived as an underexplored context, making the study’s approach unique and insightful.

In pursuit of these research goals, the paper’s organization is as follows. Section 2 provides an overview of the background and develops the hypotheses underlying the study. Section 3 outlines the methodology employed. Section 4 is dedicated to the presentation and discussion of the findings. Finally, Section 5 encapsulates the primary conclusions drawn from this study.

## 2. Hypotheses development

### 2.1. ESG

Sustainable development policy involves engaging with stakeholders to manage a company’s long-term performance while considering environmental, economic, and social factors. Corporate social responsibility (CSR) theories suggest that adopting socially and environmentally responsible practices can reduce production costs and enhance stakeholder relationships, leading to a competitive advantage and improved financial performance in the long run. The resource-based view supports this connection by emphasizing how companies can strategically leverage their resources, such as unique environmental capabilities, to gain a competitive edge and boost their financial standing. ESG reporting plays a crucial role in demonstrating a company’s commitment to its sustainability [34].

Various theories explain the motivation behind ESG reporting, including institutional theory, which suggests that companies are influenced by their institutional environments, shaped by social norms and expectations. ESG reporting aligns with these norms, contributing to social acceptance and, consequently, financial success [35]. Stakeholder theory also indicates that companies should prioritize the interests of their stakeholders, including in their commitment to sustainable development. ESG reporting demonstrates this commitment, fostering stakeholder satisfaction and ultimately enhancing financial performance [36]. In addition, legitimacy theory argues that companies operate under social contracts through which they gain and maintain social approval. Therefore, ESG reporting serves as a means to explain and justify their activities, uphold their legitimacy, and foster continued support [37].

In summary, ESG reporting serves as a critical tool for companies to demonstrate their commitment, manage stakeholder relationships, and maintain legitimacy in the eyes of society. This, in turn, contributes to their long-term sustainability and financial success.

### 2.2. Effects of ESG on corporate dividend policy

Agency theory argues that excessive cash flow paired with attractive investment opportunities (positive NPV) can incentivize managers to overinvest [38]. This may lead to companies growing beyond an optimal size, aligning with empire-building theories [39] and expanding managerial control and their potential compensation. However, these principles of overinvestment can also extend to non-financial objectives. Managers might overspend on environmental and social initiatives to gain personal benefits at shareholders’ expense, bolstering their reputation as responsible stakeholders. In [40,41], the authors linked excessive corporate philanthropy to agency costs due to its value-destroying potential. In this context, dividend policy can be a tool for discouraging overinvestment in ESG initiatives and combating agency problems. Therefore, companies with high ESG scores are expected to pay higher dividends.

Environmental, social, and governance (ESG) factors are increasingly being considered by corporations and policymakers as important determinants of corporate financial performance. ESG factors are non-financial variables that can impact a company’s risk profile, reputation, and long-term sustainability [19]. Many studies have examined the association between corporate sustainability and financial performance, and most have found that sustainability has a positive impact on financial performance [42–47]. This is probably due to factors such as reduced risk premiums, improved relationships with investors, and increased reputation. However, in [48–51], the authors argued that corporate sustainability activities are costly and that corporations have limited resources. Their studies showed that sustainability has an adverse association with firm performance. For instance, [52,53] found that ESG standards nonlinearly impact financial performance, implying that ESG standards change financial performance from positive to adverse.

One of the key areas where ESG factors are being examined is their impact on corporate dividend policy [4]. Dividend policy is a company’s decision about how much of its profits to distribute to shareholders as dividends. Traditionally, dividend policy has been seen as a financial decision based on factors such as profitability, cash flow, and investment opportunities. However, there is growing evidence that ESG factors can also play a role in a company’s dividend policy [4–7].

The association between ESG and dividends can theoretically be explained from both the shareholder value perspective and the stakeholder perspective. The shareholder value perspective argues that companies should maximize shareholder value by focusing on generating profits and distributing those profits to shareholders in the form of dividends. This perspective suggests that companies with a strong ESG performance are more likely to attract investors and have lower costs of capital, which can lead to higher profits and higher dividends. The stakeholder perspective argues that companies should consider the interests of all stakeholders, including shareholders, employees, customers, suppliers, and the environment. This perspective suggests that companies with strong ESG performance are more likely to have loyal customers, engaged employees, and strong relationships with other stakeholders. These factors can lead to long-term sustainability and profitability, which can then support a sustainable dividend policy [5–52].

There is a growing body of empirical evidence that has examined the link between ESG and dividend policy. A previous study [5] found that high-CSR companies pay out more dividends than low-CSR companies. The study also found that the relationship between CSR and dividend payout is stronger for companies with lower levels of institutional ownership in the US companies between 1991 and 2012. Another study by Ghoul et al. (2011) found that companies with higher levels of corporate social responsibility (CSR) have lower costs of capital. The study suggests that this is because investors perceive CSR companies to be less risky, as they are more likely to manage environmental, social, and governance risks effectively. Moreover, the authors of [5–52] found a positive relationship between the performance of corporate social responsibility (CSR) and dividends. For instance, Cheung et al. [6] found that companies with high CSR scores pay higher dividends because they have lower levels of perceived risk and better relationships with stakeholders. Samet and Jarboui [53] found that high CSR companies increase dividend pay-out levels. ESG practices complement the practice of rewarding shareholders, rather than diminishing its importance. By reducing conflicts of interest, minimizing information gaps, and projecting a positive image to financial markets, ESG practices enhance shareholder value [4–10]. Based on the above discussion, we propose our first hypothesis as follows:

*H1*: *There is a positive association between investment ESG and dividend policy*.

### 2.3. Moderating influence of financial sustainability on ESG and dividend policy

Financial sustainability, defined as a firm’s ability to generate long-term financial stability, plays a pivotal role in shaping ESG practices. Companies with strong financial foundations are better equipped to invest in ESG initiatives, adopt sustainable practices, and comply with environmental and social regulations [54]. Financial sustainability is also a critical determinant of dividend policy. A financially sound firm is more likely to have consistent cash flows, enabling it to sustain a regular dividend payout. Conversely, financially unstable companies may face pressure to conserve cash to address debt obligations or cover operational deficits, potentially leading to dividend cuts or suspensions [4–10].

Financial sustainability can influence the association between ESG practices and dividend policy. In detail, companies with strong financial sustainability have more resources available for both ESG initiatives and dividend payouts. This allows them to balance their commitment to ESG goals with the need to provide shareholder returns [13]. In addition, companies with strong financial sustainability are often viewed as more reliable and less risky, which can make them more attractive to investors seeking steady dividends [13–55]. Furthermore, regulatory changes related to ESG may have a significant impact on companies’ financial sustainability and dividend policy. For example, government incentives for sustainable practices may encourage companies to invest in ESG initiatives, potentially leading to higher long-term returns and increased dividend payouts [56].

In light of the discussion above, we argue that financial sustainability could influence the relationship between ESG and dividend policy. A financially sustainable firm is better positioned to reap the benefits of ESG practices, such as improved risk management, enhanced reputation, and increased investor confidence, which can support a sustainable dividend policy. We therefore propose our second hypothesis as follows:

*H2*: *Financial sustainability enhances the positive association between ESG and dividend policy*.

## 3. Materials and methods

### 3.1. Sample construction

The principal objective of this investigation is to analyze the impact of ESG practices on dividend policy, with a particular emphasis on organizations committed to financial sustainability. Our research commences with an extensive initial sample, encompassing all companies listed on the Saudi Stock Exchange’s principal market, Tadawul. The selection of our dataset was meticulous, emphasizing precision and integrity. We adopted a purposive sampling approach, deliberately excluding financial institutions and enterprises from our analysis due to the distinctive impact of regulatory considerations on their dividend patterns. Financial institutions often operate under exacting regulatory frameworks, and their financial reporting practices differ significantly from non-financial companies. This method aims to ensure a more cohesive and relevant dataset for our study, concentrating on non-financial companies with comparable characteristics. We retained observations containing essential data components that were necessary for constructing the different variables integral to our research framework. Companies with incomplete data or outliers were also eliminated, resulting in a final sample comprising 332 firm-year observations (38 companies) for the years spanning from 2013 to 2022.

### 3.2. Measurement of variables

#### 3.2.1 Measuring ESG

In this analysis, we used the extensive Bloomberg database to determine ESG performance. The Bloomberg ESG score, a methodology that assigns ratings to companies depending on the amount of their ESG disclosure, served as the foundation of our ESG review. This score is painstakingly calculated from a set of weighted data points, each tailored to the unique ESG component it represents. ESG scores are assigned on a scale of 0 to 100. A score of 0 means no or minimal disclosure, while a perfect score of 100 denotes companies who disclose every data point compiled by Bloomberg, resulting in full disclosure. Notably, our methodology is consistent with other research findings [33–59].

A total of the ESG scores were considered in our main study. For additional analysis, we exploited the three subcomponents of the ESG scores: environmental, social, and governance. Then, we gauged the incremental impact of each ESG factor on the payout ratio as a result. Additionally, information about each company’s ESG scores was gathered from the Bloomberg database.

#### 3.2.2. Measuring dividend policy

In our examination of dividend policy, we followed the methodology introduced by [11–60]. This involves the utilization of ’dividend yield,’ a metric derived by dividing the dividends per stock by the current stock price.

#### 3.2.3. Measuring financial sustainability

Selecting a tool that aids in policy research and business decision-making is crucial for assessing a company’s financial security. The sustainable growth rate (SGR) of companies is a major concern for both company management and investors, as it reflects investor expectations regarding operational stability [61]. Organizations utilize the sustainable growth rate by combining operational factors (asset efficiency and profit margin) and financial factors (retention rate and capital structure) into an essential, comprehensive indicator [62]. Various methods exist for assessing corporate sustainable growth [63,64], with the sustainable growth rate model proposed by [65] gaining widespread acceptance in previous research studies (Mukherjee and Sen, 2018). In line with other studies [66–69], we computed the sustainable growth rate derived from Higgins’ model calculation (return on equity multiplied by retention ratio). An essential measure of a company’s financial performance combines operational factors like profit margin and asset efficiency with financial components such as capital structure and retention ratio.

#### 3.2.4. Measuring control variables

Company factors can affect ESG disclosure and dividend policy. Therefore, we took into account the following control factors in accordance with numerous past studies [70–73]:

- The natural logarithm of total assets, which is used to calculate the size of a company (SIZE);
- Return on assets, which is calculated by dividing net profit by all assets (ROA);
- Financial leverage, which is determined by dividing all debt by all assets (LEV);
- Stock price volatility divided by market index volatility, which is a common way to quantify company risk (BETA);
- The natural logarithm of the company age, which is the measurement of company age (AGE).

### 3.3. Model development

**Fig 1:**
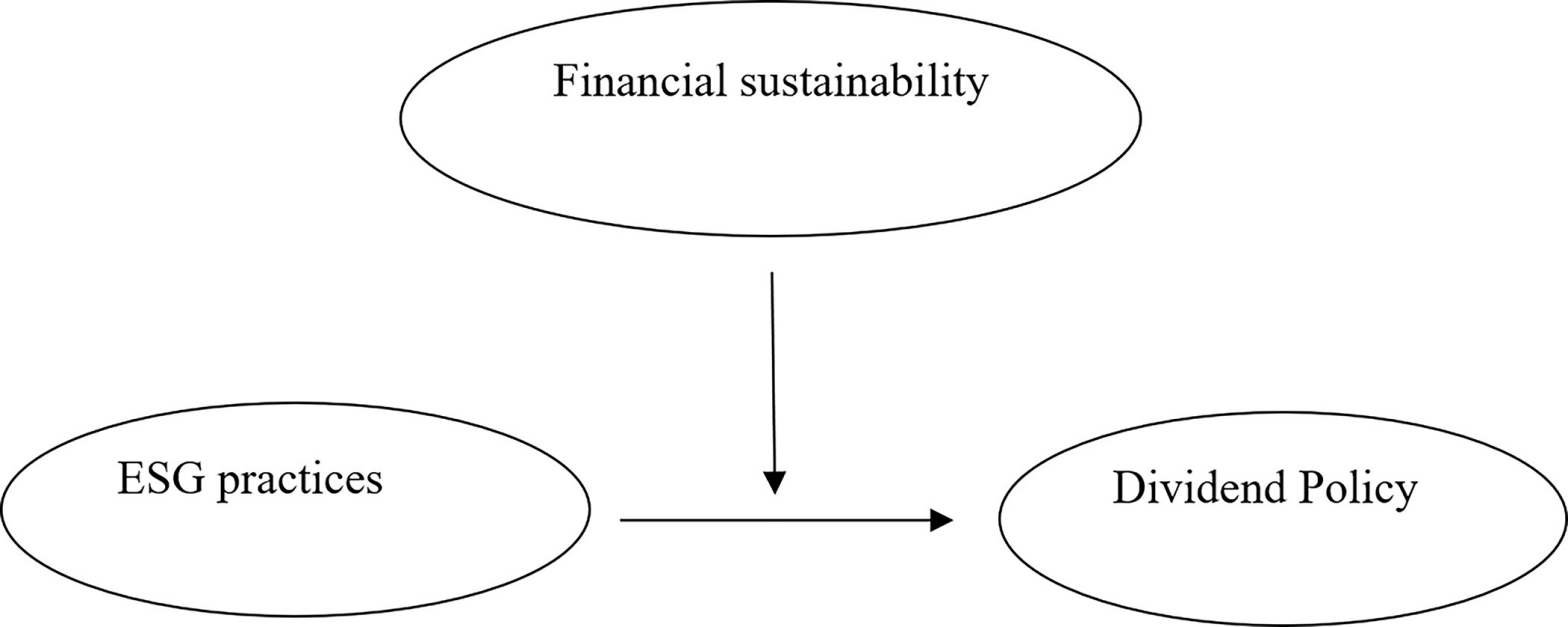
Conceptual model. Note: This figure displays the moderating effects of financial sustainability on the relationship between ESG practices and divided policy.

The expected links between ESG disclosure and dividend yields are examined in this study, along with the mediating effect of financial sustainability as displayed in Fig 1. We created the two models listed below to test our hypotheses. Our first hypothesis (HI) was tested by determining the direct impact of our predictor variable (ESG) on the dependent variable (DIV), as shown in Eq (1):

$$ESG_{i,t}=\alpha_{0}+\alpha_{1}\;DIV_{i,t}+\alpha_{2}\;SIZE_{i,t}+\alpha_{3}\;ROA_{i,t}+\alpha_{4}LEV_{i,t,}+\alpha_{5}\;BETA_{i,t}+\alpha_{6}\;AGE_{i;t}+Industry\;dummies+Year\;dummies+\varepsilon_{i,t}$$ (1)

Our second hypothesis (H2) was further supported by a moderator and interaction term, which serve to assess the moderating task of financial sustainability (SUSTAIN) on the direct connection between ESG and DIV and are represented as follows in Eq (2):

$$ESG_{i,t}=\alpha_{0}+\alpha_{1}\;DIV_{i,t}+\alpha_{2}\;SUSTAIN_{i,t}+\alpha_{3}\;DIV_{i,t}*SUSTAIN_{i,t}+\alpha_{4}\;SIZE_{i,t}+\alpha_{5}\;ROA_{i,t}+\alpha_{6}\;LEV_{i,t,}+\alpha_{7}\;BETA_{i,t}+\alpha_{8}\;AGE_{i;t}+Industry\;dummies+Year\;dummies+\varepsilon_{i,t}$$ (2)

In the equation, i and t are company and time, respectively. The Bloomberg ESG score ranges from 0 (null disclosure) to 100 (full disclosure). DIV is the ratio of dividends per share to the current share price. SUSTAIN is calculated as the ROE multiplied by the retention rate. Company size (SIZE) is the natural logarithm of total assets. Firm value is the return on assets (ROA). Financial leverage (LEV) leverage is determined by dividing all debt by all assets. Company risk (BETA) is share price volatility divided by market index volatility. Enterprise age (AGE) is the natural logarithm of company age. The terms "industry dummies" and "year dummies" refer to the commonly overlooked industry fixed effects as well as time-specific impacts that apply to all organizations and are time-variant.

Therefore, Eqs (1) and (2) were first evaluated using pooled ordinary least squares (OLS) with highly robust standard errors adjusted for heteroskedasticity and clustering by firm in order to take into consideration the lack of independence in observations within a given business over time. Fixed effects regression was used to more accurately estimate these equations and take into consideration company-specific effects that are time-invariant.

## 4. Results, analysis, and discussions

### 4.1. Descriptive statistics and Pearson’s correlation

Table 1 furnishes an overview of the statistical data pertaining to the companies in our dataset. The results illustrate a mean dividend of 29.65 with a standard deviation of 31.57 indicating that, on average, companies pay out approximately 29.65% of their earnings as dividends to shareholders. Some corporations are more generous with dividends, while others are more conservative, or choose not to pay dividends at all.

**Table 1:** Summary of statistical information.

*Variables*	N	Mean	STD	Min	Max	25th percentile	Median	75th percentile
*Div*	332	29.65	31.57	0	100	0	23.05	51.62
*ESG*	332	19.64	12.08	0.01	61.34	10.96	15.78	25.71
*ENV*	273	12.35	16.33	0	81.42	0.33	4.06	19.82
*SOC*	311	14.47	12.92	0	61.66	6.40	11.66	21.66
*GOV*	312	47.11	17.60	0	87.35	39.28	44.64	54.89
*SIZE*	332	10.71	0.74	9.23	13.27	10.23	10.70	11.25
*ROA*	332	4.06	6.78	-14.81	36.67	1.04	2.06	5.12
*LEV*	332	22.41	19.57	0	80.92	5.19	15.98	38.04
*BETA*	332	0.91	0.32	0.04	1.59	0.81	0.98	1.12
*AGE*	332	1.45	0.31	0.47	1.96	1.19	1.56	1.64
*SUSTAIN*	332	6.13	10.64	-46.6	54.9	1.37	4.98	10.21

Note: This table reports the descriptive statistics of all variables used in this study.

The average ESG score is 19.64% with a standard deviation of 12.08. ESG scores vary between 0.01 and 61.34, with a median of 15.78. These results are in line with some prior evidence obtained from the Saudi market [59]. This finding suggests that our 38 Saudi companies are not creatively allocating their environmental, social, and governance expenses to operational ESG practices because the ESG score is determined as a weighted average score ranging from 0 to 100. The voluntary nature of ESG disclosure in Saudi Arabi may possibly be a contributing factor in the low average score for ESG. For the purposes of individual ESG elements, the ENV is 12.35%, SOC is 14.47%, and GOV is 47.11%.

The average SUSTAIN value is 6.13%, indicating that Saudi enterprises have a relatively low level of financial sustainability. Therefore, this average value suggests that Saudi enterprises can achieve a maximum financial sustainable growth of 6.13% without external finance. Additionally, the average indicates that the capacity of our sample organizations for internal funding-based growth is 6.13%. In other words, businesses that require growth above 6.13% must raise more money from other sources. Furthermore, the average control variables were SIZE (10.61), ROA (4.06), LEV (22.41), BETA (0.91), and AGE (1.45).

The correlation analysis in Table 2 demonstrates that the variables ESG and DIV have positive and statistically significant correlation coefficients. We frequently ran into issues with multicollinearity in the dataset while performing multiple regression analysis. However, the correlation coefficients between each variable in our analysis offer assurance that multicollinearity problems are not present. This is because, as proposed by [74], none of the coefficients between variables exceed the predetermined cutoff value of 0.70. We estimated the variance inflation factor (VIF) to further demonstrate the absence of multicollinearity. Although they are not provided in this context, the comprehensive VIF results are available upon request. These findings show that multicollinearity is not present, as shown by the VIF values for each variable being comfortably within the permissible range (VIF 10), as suggested by [75]. As a result, we can state with confidence that our study does not have multicollinearity problems.

**Table 2:** Matrix of correlations.

Variables	Div	ESG	ENV	SOC	GOV	SUSTAIN	ROA	SIZE	LEV	BETA	AGE
Div	1										
ESG	0.094**	1									
ENV	0.086**	0.473	1								
SOC	0.055*	0.494**	0.762	1							
GOV	0.143**	0.641	-0.046***	-0.011	1						
SUSTAIN	-0.167**	-0.068	0.069	-0.057	-0.087	1					
ROA	-0.332*	0.088	0.013**	-0.009	0.081	0.302	1				
SIZE	-0.031	0.184	0.170	0.109	0.075	0.338	-0.062	1			
LEV	-0.278***	0.179	0.164	0.267**	-0.031	-0.317	-0.106	-0.433	1		
BETA	-0.175	0.024**	-0.004	0.034**	0.069	-0.208*	-0.076**	-0.677	0.130	1	
AGE	0.160	0.095	0.007	0.073	0.162	0.194	0.106**	0.331*	-0.202	-0.087	1

Note: The use of asterisks **, **, and *** indicates statistical significance at the 10%, 5%, and 1% levels, in their specific order.

### 4.2. Multivariate analysis

#### 4.2.1. Pooled OLS vs. random effects vs. fixed effects

To examine our hypotheses, we utilized panel data and employed suitable econometric methodologies. Initially, we needed to decide between three categories of panel models: the pooled OLS model, the random effects model (RE), and the fixed effects model (FE). In the process of selecting the appropriate model type, the initial step involved making a choice between the pooled OLS and random effects (RE) models through the application of Breusch–Pagan Lagrange multiplier (LM) test. If we determined that the RE model was more suitable, we could then utilize the Hausman test for random effects to make a choice between the RE and FE models. However, if we determined that the pooled OLS model is more appropriate, the subsequent decision entailed choosing between the FE and OLS models, utilizing the F-test.

Therefore, to navigate the choice between random effects (RE) and pooled ordinary least squares (OLS) models, we initially employed the Breusch–Pagan Lagrange multiplier (LM) test, as presented in Table A1 in S1 Appendix. The table indicates a probability (Prob > chibar2) below 0.05, prompting the rejection of the null hypothesis and suggesting nonzero variances across the companies, favoring the adoption of the random effects (RE) model. Subsequently, as the Breusch–Pagan Lagrange multiplier test indicates the superiority of the RE model over the OLS model, our subsequent step involved comparing the RE model with the fixed effects (FE) model. This comparison was executed through Hausman testing, as outlined in Table A2 in S1 Appendix. Within the same appendix, Table A1 in S1 Appendix demonstrates a probability (Prob > chi2) below 0.05, leading to the null hypothesis rejection. This rejection implies a correlation between the error term and the regressors, suggesting that the fixed effects (FE) model is the preferred choice in this context.

Following this model selection approach, the subsequent sections of the paper will center on the fixed effect model as the chosen preference.

#### 4.2.2. Fixed effects preliminary assumptions

To ensure the precision of a fixed effects model, certain assumptions must undergo testing. The absence of homoscedasticity, serial correlation, and the absence of cross-sectional dependence are key among these assumptions.

A critical presumption in the application of regression models to data is homoscedasticity, which denotes uniform variability among all observations. On the other hand, heteroscedasticity, which denotes non-constant variance, is the term used when the dispersion differs between observations. Regression coefficient estimators are affected by heteroscedasticity; however, they are still unbiased in the presence of heteroscedasticity [76]. We applied the Breusch-Pagan to check for heteroscedasticity in the panel data. The results shown in Table A3 in S1 Appendix show that the Prob > chi2 (0.000) is below 0.05. Consequently, we can reject the null hypothesis and infer the existence of heteroscedasticity in our panel data.

Detecting serial correlation in linear panel-data models is crucial, as it introduces bias in standard errors and diminishes result efficiency. Researchers strive to identify such serial correlation in the idiosyncratic error term of a panel-data model. The Wooldridge test is particularly appealing for this purpose, given its minimal assumptions and straightforward implementation [77]. The results of the Wooldridge test are presented in Table A4 in S1 Appendix, revealing that the Prob > F (0.0530) exceeds 0.05. Therefore, we can accept the null hypothesis and affirm that there is no evidence of serial correlation in our panel data.

An increasing body of work in the literature on panel data indicates that panel datasets are prone to significant cross-sectional dependence. This dependency may be attributed to common shocks and unobserved components that become integrated into the error term. Additionally, spatial dependence and idiosyncratic pair-wise dependence in the disturbances, without a discernible pattern of common components or spatial relationships, contribute to this phenomenon [78]. Pesaran’s CD-test is employed to examine cross-sectional dependence in panel data, as reported in [79]. The results, shown in Table A5 in S1 Appendix, indicate p-values exceeding 0.05, leading to the acceptance of the null hypothesis and the conclusion that there is no evidence of cross-sectional dependence in our panel data.

Given the violation of the homoscedasticity assumption, we opted for a fixed effects model with robust standard errors to address this deviation and ensure the accuracy of our analysis.

### 4.3. Results

#### 4.3.1 The impact of ESG on dividend

This section presents empirical findings on the impact of ESG disclosure on the dividend policy of companies listed on the Saudi Exchange. Table 3 demonstrates that the model of the fixed effects regression with robust standard errors supports our first hypothesis (H1). The findings claim a positive and significant association between total ESG and payout ratio as determined by DIV. According to the statistical significance of the link at the 1% level, increased disclosure of company ESG practices are thought to increase dividend distribution.

**Table 3:** The impact of ESG on dividend.

VARIABLES	Expected sign	Fixed Effects with robust standard errors
Intercept	-/+	93.4781*** (2.67)
ESG	+	0.5714*** (3.41)
SIZE	-/+	-6.5781** (-2.10)
ROA	-/+	0.1863 (0.57)
LEVERAGE	-	-0.3914*** (-3.73)
BETA	-/+	1.3150 (0.24)
AGE	+	4.7768 (0.76)
Year_FE		Yes
Industry_FE		Yes
Observations		320
F_statistic		2.80***
Adjusted R^2^		0.0731

Note: The use of asterisks **, **, and *** indicates statistical significance at the 10%, 5%, and 1% levels, in their specific order.

With respect to the control variables, our findings show that SIZE is negatively and significantly related to the DIV. This finding supports the view that larger companies are less likely to pay dividends than smaller ones. ROA is inversely and significantly associated with Div, also indicating that high-performance companies are less likely to transfer dividends to their shareholders. LEV is also negatively and significantly associated with a DIV, which signifies that highly leveraged companies dispense less dividend. For the RISK variable, the results show that when a company is associated with a higher risk it has an adverse and significant impact on payout ratio. Finally, the older companies (AGE) are positively and significantly related to the DIV. These results suggest that a company’s dividend yields are related to its characteristics.

#### 4.3.2. The role of financial sustainability on the association between ESG and dividend

Table 4 shows the results of the fixed effects analysis with robust standard errors for the regression of SUSTAIN (moderating variable) on the association between ESG and DIV. The estimated coefficient of SUSTAIN is positive and significant, proposing that the new corporate financial sustainability increases the payout ratio of Saudi companies. The anticipated coefficient of SUSTAIN*ESG is statistically significant and positive, suggesting that corporate financial sustainability enhances the positive impact of ESG disclosures on dividend policy. This result supports our second hypothesis (H2), which states that long-term performance of a firm has a positive moderating influence on the association between ESG activities and dividend policy.

**Table 4:** The role of financial sustainability on the association between ESG and dividend.

VARIABLES	Expected sign	Fixed Effects with robust standard errors
Intercept	-/+	54.6012* (1.76)
ESG	+	0.3584** (2.33)
SUSTAIN	-/+	1.4773*** (2.18)
ESG*SUSTAIN	-/+	0.0269** (4.69)
SIZE	-/+	-2.1069 (-0.75)
ROA	-/+	1.7413*** (5.16)
LEVERAGE	-/+	-0.4980*** (-5.17)
BETA	-/+	-3.7203 (-0.75)
AGE	-/+	5.9809 (1.07)
Year_FE		Yes
Industry_FE		Yes
Observations		320
F_statistic		12.67***
Adjusted R^2^		0.2265

Note: The use of asterisks **, **, and *** indicates statistical significance at the 10%, 5%, and 1% levels, in their specific order.

## 5. Additional analysis

### 5.1. The effects of environmental disclosure, social disclosures, and governance disclosures on dividend policy

Table 5 reports the regression of separate ESG constituents, ENV, SOC, and GOV, on dividend yields utilizing fixed effects regressions with robust standard errors. First, the predicted coefficient of ENV in is positive and statistically significant (at the 10% level), meaning that greater levels of disclosed environmental data lead to higher corporate financial sustainability. This finding is similar to that in [13]. Furthermore, a remarkable result shows a significant and positive link between social transparency and dividend payments, albeit at a 1% significance level. This result is in perfect agreement with the conclusions reached in [80]. Corporate Social Responsibility practices are viewed as a sign of a company’s unwavering duty to the community. This ultimately improves the firm’s standing and reputation in the financial market, that consequently strengthens its financial value and makes dividend payments easier. The corporate governance measures have a very beneficial effect on the propensity to issue dividends, as demonstrated by the governance constituent of ESG index. This suggests that these governance measures function well in a setting known for robust investor safeguards and well-established stock exchanges. By encouraging the long-term survival of enterprises and protecting shareholders’ welfare with a liberal dividend approach, they play a dual role, according to the research in [81].

**Table 5:** The impact of environmental disclosure, social disclosure and governance disclosure on dividend policy.

VARIABLES4.3.	ENV	SOC	GOV
	Fixed Effects (1)	Fixed Effects (2)	Fixed Effects (3)
Intercept	126.3452*** (3.13)	63.5599 (0.97)	94.7484*** (2.66)
ENV	0.1766* (1.76)		
SOC		0.4924*** (2.71)	
GOV			0.3318*** (3.24)
SIZE	-8.7864** (-2.3)	-0.5499 (-0.09)	-7.1806** (-2.24)
ROA	1.1907*** (3.00)	-0.6995** (-2.13)	0.2122 (0.69)
LEVERAGE	-0.4500*** (-3.90)	0.1269 (0.57)	-0.3568*** (-3.32)
BETA	3.2963 (0.61)	5.8851 (1.21)	-1.8791 (-0.34)
AGE	2.5617 (0.39)	21.8617 (-1.23)	4.2020 (0.66)
Year_FE	Yes	Yes	Yes
Industry_FE	Yes	Yes	Yes
Observations	266	299	305
F_statistic	7.25***	2.80**	5.79***
Adjusted R^2^	0.1239	0.1315	0.0865

## 6. Robustness checks

### 6.1. Alternative methods of estimation

We have modified our estimating methods to consider both cross-sectional and serial dependencies, much as in [82,83]. To further study the impact of ESG and payout ratio, several assessment models are presented in Table 6. Feasible generalized least squares (FGLS) is used in Model (1), whereas the Fama–MacBeth approach is used in Model (2). Models (3) and (4) are built using the technique of [84] and quantile regression, respectively. Finally, the Driscoll–Kraay robust standard error model is used to implement Model (5). Our choice of estimation techniques is aligned with the distinctive characteristics of our study on the impact of ESG disclosures on dividend policy. The selection of feasible generalized least squares (FGLS) is driven by its robustness in handling both heteroscedasticity and serial correlation. Fama–MacBeth is employed to capture the dynamics of time-series cross-sectional data, while the Newey–West estimator addresses potential biases due to heteroscedasticity and autocorrelation. Quantile regression allows us to explore variations across different quantiles, providing a nuanced perspective. To account for spatial correlation, the Driscoll–Kraay robust standard error model is utilized.

**Table 6:** The impact of ESG disclosures on dividend policy employing different estimation techniques.

VARIABLES	FGLS	Fama-MacBeth	Newey-West	Quantile	Driscoll-Kraay robust standard error model
Intercept	86.2671** (2.08)	111.2328* (3.12)	98.7161*** (2.86)	38.0330*** (2.78)	57.1081** (2.94)
ESG	0.4753*** (3.13)	0.8819* (4.14)	0.4879*** (3.31)	0.5787** (2.44)	0.4313*** (3.98)
SIZE	-5.9724 (-1.60)	-8.7474 (-2.80)	-7.0522** (-2.34)	-1.0501 (-0.25)	-3.3229 (-1.81)
ROA	0.2878 (0.71)	0.4019 (1.05)	0.3491 (0.93)	1.2552*** (2.69)	0.3755 (1.19)
LEVERAGE	-0.3857*** (-3.46)	-0.3269 (-2.08)	-0.3429*** (-3.24)	-0.5335*** (-3.26)	-0.5016** (-2.31)
BETA	1.1699 (0.27)	-5.1102 (-1.67)	-2.4119 (-0.54)	-1.6351 (-0.19)	1.7713 (0.26)
AGE	3.5969 (0.57)	6.6013 (1.08)	4.1966 (0.67)	-2.8786 (-0.29)	6.8783 (0.70)
Year_FE	Yes	Yes	Yes	Yes	Yes
Industry_FE	Yes	Yes	Yes	Yes	Yes
Observations	320	332	332	332	320
Adjusted R^2^	0.1020	0.1408	0.045	0.134	0.2785

Note: The use of asterisks **, **, and *** indicates statistical significance at the 10%, 5%, and 1% levels, in their specific order.

### 6.2. Tracking endogeneity (GMM)

The current findings suggest that ESG has a positive impact on dividends. However, the observed positive association may be influenced by potential endogeneity problems, in which certain unobservable factors affecting both ESG and dividends may be at work. To address this issue, we ran a test using a dynamic panel GMM technique, with the goal of mitigating potential endogeneity concerns and improving the robustness of our study.

Table 7 shows the empirical results of the two-step generalized method of moments (GMM) used to address endogeneity issues. In terms of the first-order autocorrelation of errors and the lack of autocorrelation of second-order residuals, the AR1 and AR2 checks support the rejection of the alternative hypothesis. This means that we reject the autocorrelation alternative hypothesis.

**Table 7:** Tracking endogeneity (GMM).

VARIABLES	GMM
INTERCEPT	18.5657 (0.62)
LAG DIV	0.6117*** (3.06)
ESG	0.0906*** (2.62)
SIZE	-1.4321 (-0.53)
ROA	0.5798** (2.22)
LEVERAGE	-0.1849 (-1.32)
BETA	2.1284 (0.39)
AGE	5.2138 (1.03)
N	247
AR(1) test (p-value)	-0.73**
AR(2) test (p-value)	-1.90
Hansen-J test of over-identification (p-value)	3.86
Year	Yes
Industry	Yes

We validated our previous results by showing a positive influence of ESG on dividends distribution.

## 7. Discussion

### 7.1 The impact of ESG on dividend

The positive impact of environmental, social, and governance (ESG) factors on dividends in the Saudi context mirrors findings in other diverse contexts [85–87]. Various plausible explanations exist for this phenomenon. Companies dedicated to sustainability appear to not only cultivate greater financial resilience but also generate significant excess cash. This surplus, arising from a consolidated business approach, acts as a catalyst for positive transformation. A substantial body of work in the literature and empirical evidence both emphasize that organizations with excess cash are better positioned to invest in ESG-related practices [88,89]. This strategic allocation of resources not only reinforces the company’s sustainability commitment but also augments shareholder value. As the ripple effect of these investments unfolds, it culminates in the rewarding distribution of dividends to shareholders, illustrating the interconnected relationship between sustainability, financial strength, and shareholder returns [13].

Furthermore, heightened transparency in disclosure practices tends to correlate with increased dividend distributions [13–91]. The focus on paying dividends remains paramount, and engaging in ESG practices does not diminish the importance of rewarding shareholders. Instead, it serves to mitigate agency conflicts, reduce information imbalances, and send favorable signals to the financial markets [4–10].

Thus, the current study provides valuable insights into the potential benefits of integrating ESG practices with financial strategies, promoting sustainability, and enhancing shareholder returns in the Saudi context.

Our findings about the positive effect of ESG on distributing dividends lead to significant practical implications for businesses. Companies that prioritize and integrate ESG considerations into their operations can potentially enhance their corporate reputation, appealing to stakeholders who increasingly value ethical and responsible business practices. This positive association may attract socially conscious investors and contribute to building trust with customers and employees. Furthermore, a commitment to ESG principles can serve as a strategic tool for risk management, as companies that actively address environmental and social concerns may be better positioned to navigate regulatory changes and mitigate reputational risks. Additionally, the positive correlation with dividend policy suggests that financial success and sustainability initiatives need not be mutually exclusive. Companies can view ESG practices not only as a means of fulfilling corporate social responsibility but also as an avenue for long-term financial viability and value creation for shareholders.

### 7.2 The role of financial sustainability on the association between ESG and dividend

This study shows that financial sustainability measured by the sustainable growth rate enhances the positive effect of ESG on dividend distributions. In fact, financially sustainable businesses are becoming more prevalent. Businesses are increasingly investing in ESG practices purposefully and strategically. According to [22], this dedication to ESG is more than just a fad, it is a deliberate attempt to build a favorable reputation and foster confidence among their stakeholders. ESG practices are now a crucial way of demonstrating how committed a firm is to social responsibility, environmental responsibility, and sound governance principles in today’s business environment. Businesses hope to obtain the respect and confidence of their investors and the general public by implementing these sustainable practices.

In addition, we discovered that the payment of dividends to investors is a top priority for companies with strong financial resilience. Their steadfast dedication to the people and organizations who have entrusted them with their resources is represented by their commitment to timely dividend payments. These businesses consider dividends as more than just money exchanges; they stand for a significant promise and a solid link between the company and its investors, as stressed by [92]. This steadfast dedication to dividend payments is a monument to their financial stability and their understanding of the value inherent in repaying their stakeholders for their confidence and investment in the expansion and success of the company. Thus, the positive effect of ESG activities on dividend policy is more pronounced in financially sustainable companies.

Overall, the sustainable growth rate serves as a comprehensive metric, encompassing not only financial performance but also a company’s ability to balance economic growth with environmental and social responsibilities. When financial sustainability aligns with ESG practices, it creates a synergistic effect, indicating that companies not only prioritize financial robustness but also actively invest in initiatives that contribute positively to the environment, society, and governance [93]. This alignment not only signals a commitment to responsible business practices but also enhances stakeholder perception and mitigates long-term risks. This study implies that the combination of financial sustainability and ESG practices contributes to the creation of enduring value for shareholders, reflecting a strategic and holistic approach to business that considers financial health, sustainability, and stakeholder value creation in tandem.

These results have significant practical implications for businesses. They suggest that those companies strategically embracing both ESG initiatives and financial sustainability can optimize resource allocation, enhancing the positive impact on dividend distribution. Then, this dual commitment becomes a compelling value proposition for investors seeking a blend of ethically and financially sound investments, thereby boosting investor confidence. Moreover, understanding this moderation effect allows companies to manage risks effectively by concurrently focusing on financial stability and responsible business practices. This approach contributes to long-term value creation, reinforcing a corporate identity that aligns with stakeholder expectations and regulatory standards. Leveraging this understanding can enhance corporate reputation, appealing to stakeholders who prioritize sustainability goals alongside financial returns. In essence, this positive moderating effect underscores the strategic advantage of aligning financial sustainability with ESG practices, creating a resilient, responsible, and value-driven corporate identity with far-reaching positive implications for investor relations, risk management, and overall business sustainability.

## 8. Conclusion

This study was primarily motivated by the existing gap in empirical evidence concerning the influence of ESG factors on decisions related to dividend payouts and the moderating role of financial sustainability within the ESG–dividend association, specifically within the Saudi context.

To address this need, our study contributed to this line of research by conducting a cross-country analysis, utilizing data from 38 companies listed on the Saudi Exchange covering the period of years from 2013 to 2022. In doing so, it offers both conceptual backing and empirical substantiation regarding the positive impact of ESG indices and the dividend distribution ratio. Thus, this broader examination fills a critical void in our understanding of how ESG considerations impact dividend-related decisions within the Saudi setting. Moreover, the findings provide additional insights into the fact that the connection joining ESG to dividend policy is significantly strengthened by financial sustainability. This underscores the notion that companies are dedicated not solely to their shareholders but also to their broader spectrum of stakeholders. We undertook a series of rigorous tests to affirm the robustness of our findings, employing alternative estimation methods.

This study carries several significant implications. First, the theoretical implications of this paper extend beyond the empirical findings, offering valuable contributions to the existing literature. By delving into the relationship between ESG considerations and dividend policies within the Saudi context, our study enriches the theoretical framework surrounding corporate decision-making. The identification of financial sustainability as a reinforcing factor in the link between ESG and dividend policy provides a nuanced understanding of how broader sustainability practices influence specific financial outcomes. This insight contributes to refining existing theories that explore the interplay between environmental, social, and governance factors and financial performance. Moreover, the recognition of ESG disclosure as a strategic investment underscores the evolving role of ESG considerations in corporate strategy, challenging traditional views of shareholder-centric decision-making.

Therefore, it is recommended that policymakers in Saudi Arabia should consider leveraging these findings to bolster the regulatory frameworks that encourage and incentivize companies to adopt robust ESG practices. Recognizing the positive impact of ESG performance on dividend payments, policymakers can implement measures that promote sustainability reporting standards, ensuring transparency and accountability among listed companies. Financial regulators may also find value in promoting financial sustainability as a key moderating factor in the ESG–dividend relationship. Encouraging businesses to integrate financial sustainability into their operational strategies can be incentivized through regulatory support and guidelines. Furthermore, practitioners and corporate decision-makers can use these insights to align their strategic planning, acknowledging that strong ESG practices not only contribute to sustainable development but can also enhance the reliability of dividend payments.

For managers, this suggests that cultivating and maintaining strong ESG standards not only contributes to sustainable development but also serves as a means of showcasing commitment to both stakeholders and shareholders through consistent dividend payments. Moreover, recognizing the enhancing influence of financial sustainability on the ESG–dividend relationship provides an actionable insight for managers. Companies that prioritize financial sustainability alongside ESG considerations are likely to experience a more notable positive impact on dividend yields compared to their counterparts. Therefore, managers are encouraged to align their corporate strategies to incorporate a dual focus on ESG practices and financial sustainability, thereby enhancing the overall financial performance and stakeholder value proposition of their organizations. This dual emphasis can position companies to thrive in an environment increasingly shaped by sustainable business practices and stakeholder-oriented governance.

For investors, the implications drawn from this study offer valuable insights into the intersection of ESG considerations and dividend policy within the Saudi context. The identified strong positive impact of ESG performance on dividend payments signifies a potential marker for investors seeking sustainable and socially responsible investment opportunities. Companies with robust ESG practices, as revealed by the study, not only demonstrate dedication to stakeholders and shareholders but also maintain a consistent pattern of dividend payments. This insight provides investors with a lens through which they can align their investment portfolios with socially responsible and financially rewarding companies. Moreover, the acknowledgment of financial sustainability as a moderating factor in the ESG–dividend relationship enhances the discernment for investors. Companies that integrate financial sustainability with strong ESG practices exhibit a more notable positive effect on dividend yields compared to their peers. Investors are thereby encouraged to consider not only a company’s ESG performance but also its financial sustainability as critical indicators of the company’s long-term financial stability and potential returns. This study equips investors with valuable information to make informed decisions aligned with their financial and ethical considerations in the dynamic landscape of the Saudi market.

While our study provides valuable insights, it is crucial to acknowledge certain limitations that may impact the interpretation and generalizability of our findings. Firstly, the reliance on panel data from companies listed on the Saudi Exchange might not fully capture the diverse dynamics of the global markets, so we caution against broad generalization to other settings or regions. Secondly, while financial sustainability is considered as a moderating factor, unexplored contextual variables and industry-specific dynamics could potentially influence the observed relationships. Thirdly, the use of quantitative data limits our ability to offer in-depth qualitative insights into the motivations behind companies’ ESG practices and their effects on dividend decisions. Recognizing these limitations, future research endeavors could delve deeper into these aspects for a more comprehensive understanding. Furthermore, exploring potential non-linearities and threshold effects in the ESG–dividend relationship would enhance our understanding of how companies navigate sustainability concerns and financial performance. Additionally, employing other proxies for sustainable growth rate (such as those proposed by the Boston Consulting Group: Zakon, Sparkman, Ulrich and Arlow, Johnson, Lewellen and Kracaw, Firer and Ross; and the Van Horn model) could provide a more comprehensive understanding of the moderating role of financial sustainability in the relationship between ESG considerations and dividend policies.

## Supporting information

S1 Appendix Hausman, Breusch and Pegan Lagrangian multiplier, serial correlation, heteroscedasity and cross-sectional dependence tests. (DOCX)
