# Supplementary material for: The power of ESG in shaping dividend policy: Illuminating the role of financial sustainability in an emerging market
Source: PLoS One. 2024 Dec 5;19(12):e0312290. doi: 10.1371/journal.pone.0312290 (PMC11620373; doi:10.1371/journal.pone.0312290)
Supplement: S1 Appendix — (DOCX) [file pone.0312290.s001.docx]

**Appendix A. Hausman, Breusch and Pegan Lagrangian multiplier, serial correlation, heteroscedasity and cross-sectional dependence tests.**

**Table A1: Breusch and Pagan Lagrangian multiplier test for random effects**

div[id,t] = Xb + u[id] + e[id,t]

Estimated results:

|  | **Var** | **SD = sqrt(Var)** |
| --- | --- | --- |
| Div | 1034.616 | 32.16545 |
| E | 539.6067 | 23.22944 |
| U | 410.4517 | 20.25961 |

Test: Var(u) = 0. chibar2(01) = 184.34. Prob > chibar2 = 0.0000

**Table A2: Hausman fe_model re_model**

|  | **Coefficients** | |  |  |
| --- | --- | --- | --- | --- |
| **Variables** | **(b)**  **fe_model** | **(B)**  **re_model** | **(b-B)**  **Difference** | **sqrt(diag(V_b-V_B))**  **Std. err.** |
| ESG | 0.2599075 | 0.21173 | 0.0481774 | 0.096728 |
| SIZE | 0.459807 | -2.628905 | 3.088712 | 4.426301 |
| ROA | -0.4797477 | -0.2264706 | -0.2532771 | 0.1573186 |
| LEVERAGE | -0.0555737 | -0.2316842 | 0.1758106 | 0.1551697 |
| BETA | 6.572567 | 5.070058 | 1.502509 | 1.326415 |
| AGE | -36.90386 | 2.56425 | -39.46811 | 21.37436 |

b = Consistent under H0 and Ha; obtained from xtreg. B = Inconsistent under Ha, efficient under H0; obtained from xtreg. Test of H0: Difference in coefficients not systematic. chi2(6) = (b-B)'[(V_b-V_B)^(-1)](b-B) = 13.55; Prob > chi2 = 0.0051.

**Table A3: Breusch–Pagan/Cook–Weisberg test for heteroskedasticity**

Assumption: Normal error terms

Variable: residuals

H0: Constant variance

chi2(1) = 49.08

Prob > chi2 = 0.0000

Table A4: Wooldridge test for autocorrelation in panel data

H0: no first-order autocorrelation

F(1, 37) = 3.994

Prob > F = 0.0530

**Table A5:** **Pesaran's test of cross-sectional independence** = 0.517, Pr = 0.6053
